# Towards immersive virtual reality (iVR): a route to surgical expertise

**DOI:** 10.1186/s40244-015-0015-8

**Published:** 2015-05-07

**Authors:** Saurabh Dargar, Rebecca Kennedy, WeiXuan Lai, Venkata Arikatla, Suvranu De

**Affiliations:** Center for Modeling, Simulation and Imaging in Medicine, Rensselaer Polytechnic Institute, 110 8th Street, Troy, NY 12180-3590 USA

**Keywords:** Immersive virtual reality, Haptic technology, Surgical simulations, Surgical learning

## Abstract

Surgery is characterized by complex tasks performed in stressful environments. To enhance patient safety and reduce errors, surgeons must be trained in environments that mimic the actual clinical setting. Rasmussen’s model of human behavior indicates that errors in surgical procedures may be skill-, rule-, or knowledge-based. While skill-based behavior and some rule-based behavior may be taught using box trainers and *ex vivo* or *in vivo* animal models, we posit that multimodal immersive virtual reality (iVR) that includes high-fidelity visual as well as other sensory feedback in a seamless fashion provides the only means of achieving true surgical expertise by addressing all three levels of human behavior. While the field of virtual reality is not new, realization of the goals of complete immersion is challenging and has been recognized as a Grand Challenge by the National Academy of Engineering. Recent technological advances in both interface and computational hardware have generated significant enthusiasm in this field. In this paper, we discuss convergence of some of these technologies and possible evolution of the field in the near term.

## Review

### Introduction

Performing surgery requires a broad spectrum of psychomotor, cognitive, and interprofessional skills to complete complex tasks in stressful environments. Therefore, intensive training is needed for surgeons to master techniques and attain surgical expertise. Much research has been focused on training technical skills for surgery, such as suturing and knot tying [1], resulting in standardized certification programs like the Fundamentals of Laparoscopic Surgery (FLS) curriculum, which is endorsed by the American College of Surgeons (ACS) and the Society of American Gastrointestinal and Endoscopic Surgeons (SAGES). However, technical skills are only one aspect of surgical expertise. After mastering technical skills, surgeons must combine them into complex tasks and procedures. Further, surgery takes place under stressful, attentionally demanding conditions. The surgeon must perform tasks with enough spare attention to multitask.

Traditionally, trainees have acquired surgical skills through an apprenticeship model, in which they observe senior surgeons and perform under their guidance. However, this model is inadequate for more complex procedures like laparoscopic surgery [2]. As a solution, simulations and virtual environments provide a way to train surgeons in highly realistic conditions to better prepare them for the operating room. The advantages of using virtual environments for training have been recognized for decades in aviation [3] and the military [4], but the use of virtual environments for training in healthcare is a relatively new concept. Satava [5] first proposed simulation for surgical skill acquisition in the early 1990s. In the surgery domain, as in other domains, potential benefits of simulation for training and assessment are widespread, including improved safety, cost-effectiveness, standardization, repeatability, and instructional flexibility compared to many traditional training methods [6,7]. Virtual reality as a form of simulation is especially useful for training because it provides *highly realistic* settings for individuals to ‘learn by doing’ to better prepare them for clinical settings [8,9].

While the concept of virtual reality is not new, reaching a level that is sufficiently immersive has been recognized as a Grand Challenge by the National Academy of Engineering [10]. However, recent technological advances are promising. The purpose of this article is to provide an overview of how immersive virtual environments can be used to train surgical skills; specifically, technological advancements that are enabling the development of high-fidelity multimodal immersive environments to train higher-level skills characteristic of expert surgeons.

#### Developing expertise

Surgeons develop technical skills using box trainers, observations of live or video-recorded procedures, and *ex vivo* and *in vivo* animal models. However, these training methods alone are not sufficient for trainees to attain surgical expertise. It is known in the psychology literature that for an individual to achieve expertise in a given set of skills, he or she must undergo a regimen of deliberate practice, often for 10 years or more [11]. During limited training hours with traditional methods, surgical trainees likely do not receive enough practice to achieve the expertise that comes with thousands of hours of practice with varied cases and unexpected complications.

From a cognitive standpoint, expertise is characterized by achieving *automaticity*, that is, automatic cognitive processing [12-14]. Automatic processing is fast and performed with little conscious attention, in contrast to controlled processing, which is slower and takes more cognitive effort. As expertise is achieved, the type of processing used for the tasks shifts from controlled to automatic as the individual learns sequences of events that can be carried out automatically [13,14]. For surgeons, automaticity is achieved when they have enough practice that they are able to perform technical tasks automatically, using few attentional resources and leaving spare attention available for multitasking in the operating room [15-17]. Multitasking for surgeons might include dealing with distractions or unexpected issues or monitoring information about the patient’s status. If the surgeon’s entire attentional capacity is being devoted to the psychomotor surgical task itself, as is likely the case with novice surgeons, performance might suffer in the face of distractions and interruptions that are characteristic of the operating room setting.

Virtual reality can help individuals move towards expertise and automaticity by providing the opportunity for repeated practice under conditions that closely match the real environment. Virtual environments can be immersive and highly realistic, providing training benefits beyond traditional training methods. For example, adding multimodal components to a virtual environment (e.g., sounds, haptic feedback, smells) can help trainees experience the scenario as if it were real, reducing the ‘adrenaline gap’ that is often experienced by students performing tasks in simulated environments [18]. Further, practice in these environments can help trainees gain confidence so that they *feel* better prepared for operating on actual patients. Stress-training theories suggest that individuals should be given enough training and resources to perceive themselves as competent for a given situation [19]. Realistic virtual environments can help surgeons develop high-level skills while also reducing stress and improving their confidence for carrying out those skills in the real environment, with real patients.

#### Addressing skill-, rule-, and knowledge-based behaviors with virtual environments

The use of immersive virtual environments should be considered in the context of a training curriculum. Curricula should be developed to include clear definitions of skills to be learned, methods of measurement, benchmarks for learners to achieve, and feedback to be given to learners [20,21]. Using these specifications, interactive simulation scenarios can be designed specifically to match training goals, and throughout the curricula, these goals may build towards expertise in a stepwise manner. In a paper describing the manners in which simulation should be integrated into surgical training curricula, Gallagher and colleagues [2] suggested that learning complex tasks often exceeds a surgeon’s limited attentional capacity. Just as with learning any complex skill, such as driving a car [22], surgical skills might best be learned in a stepwise manner, in which basic skills are acquired first and are eventually combined into a complete task performed in a realistic setting. Molduvanu et al. [23] also suggested using a combination of training methods to address different skills to best prepare surgeons for the operating room.

Rasmussen [24] provides a framework for describing human behaviors as skill-, rule-, or knowledge-based, which can be used as a reference when designing a training method or curriculum as detailed in Table 1. Underlying this framework is the assumption that humans are goal-oriented and seek relevant information for decision-making. Accordingly, the three levels of human behavior identified by Rasmussen [24] are skill-, rule-, and knowledge-based behaviors, which are differentiated by the strategy used to gather information and make decisions. Essentially, different strategies require different amounts of attentional resources and effort by individuals during specific tasks. This framework can be used to classify the skills to be trained and the best means for training them [25].

**Table 1:** **Rasmussen [** 24 **] provides a framework for describing human behaviors as skill-, rule-, or knowledge-based**

**Level of behavior**	**Characteristics of level**	**Training tools for level**
Skill-based behavior	-Automatic, using few attentional resources -Patterns stored in memory for well-practiced, routine tasks	-Box trainers -Virtual reality simulators that address basic skills
Rule-based behavior	-Requires some attentional resources -Rules and procedures are stored in memory -Individual decides which rules and procedures to apply to situations	-*Ex vivo* or *in vivo* animal models -Virtual reality simulators that combine basic skills into procedures
Knowledge-based behavior	-Places heavy demands on attentional resources -No stored patterns, rules, or procedures in memory -Used for novel, rare, or unique situations	-Virtual reality simulators that combine basic skills into procedures and also introduce complications like distractions, interruptions, or rare events

First, *skill-based behavior* refers to automated and highly integrated actions that can occur using few attentional resources. Behavior at the skill-based level is governed by patterns of activity stored in memory for well-practiced, routine situations [26]. *Rule-based behavior* is more goal-oriented in nature. Behavior at this level follows a set of stored rules or procedures [26], requiring an individual to direct conscious attention to recognizing a situation and retrieving appropriate rules from memory. Finally, *knowledge-based behavior* is required for unfamiliar situations, for which there are no pre-specified rules or procedures. In these situations, the individual must plan actions using conscious analytical processes [26], which places heavy demand on attentional resources [27,28].

Based on Rasmussen’s model [24], we deduce that virtual environments can be developed to address each of the behavior levels [29,30]. Existing virtual reality-based simulators can support skill-based behavior by supporting basic psychomotor skills (e.g., hand-eye coordination) and simple technical skills (e.g., suturing and knot tying). These simulators can enable practice time for elementary skills beyond what trainees could otherwise acquire during apprentice training [31,32]. Laparoscopic surgery in particular is well suited for psychomotor training using virtual environments [33,20] (because it is a complex skill requiring a lot of practice to master). A widely used laparoscopic surgery training system is the MIST VR (minimally invasive surgery trainer - virtual reality), which was introduced in the 1990s as a low-cost virtual reality trainer. The MIST VR has been widely studied in terms of learning basic skills [32]. Several studies have demonstrated that training with the MIST VR has been useful in overcoming visuospatial and psychomotor challenges inherent in performing laparoscopic surgery (e.g., [34-36]).

Beyond basic skills, immersive virtual environments can also be used for whole-task training to help individuals learn proper sequences of steps and transitions between them (i.e., rule-based behavior; [32]) or for crisis management training (i.e., knowledge-based behavior; [37-40]). As the trainee develops expertise through mastering basic skills, he or she can practice rule- and knowledge-based behavior by performing live or virtual procedures that combine basic skills into a more complete process, eventually performed under more realistic conditions. For example, stress exposure training provides an opportunity for trainees to practice performing tasks under stressful conditions similar to real-world conditions [41,42]. For developing knowledge-based behavior skills in particular, the use of training scenarios that highlight rare or unusual circumstances, or present unexpected complications, can be especially useful [22].

Immersive virtual environments might also be used to address nontechnical skills like teamwork, communication, and intercultural sensitivity, further addressing behaviors at the knowledge-based level. In particular, interactions with virtual humans afforded by virtual environments can enable safe, repetitive, deliberate practice of clinical and interpersonal skills [43,44]. For example, a virtual patient system called the Virtual Objective Structured Clinical Examination (VOSCE) has been developed with the purpose of grading medical students on patient interview skills [45-47]. The VOSCE combines life-size projections of virtual characters, head tracking, gesture recognition, and speech recognition to enable natural interactions.

Recent technological advances are enabling more immersive and realistic training experiences, better supporting training at higher levels of rule- and knowledge-based behavior. The U.S. National Academy of Engineering listed 14 Grand Challenges formulated based on human needs for sustainability, health, and joy, one of which is the challenge to enhance virtual reality. The committee who put together the list of Grand Challenges identified several advances needed in virtual reality for systems to fully simulate reality [10]. In particular, the lack of visually precise detail and the lack of realistic tactile and haptic feedback have traditionally been shortcomings of virtual environments, but technological advancements are quickly improving the capability to create high-fidelity, multimodal virtual environments.

It is indeed a sizeable challenge to virtually replicate a complex setting like an operating room. However, immersive virtual environments provide the only real avenue for fully addressing training at the knowledge-based behavior level. In the following sections, we will describe the technical and psychological aspects of immersive virtual environments that can ultimately lead to effective training of high-level surgical skills.

#### Defining immersive VR

When discussing the use of virtual environments, it is useful to step back and define different aspects of these systems. *Virtual environments* can be defined as artificial environments that are designed to appear and feel like a real environment [7]. These environments can range in the level of immersion generated, impacting the degree to which a user perceives the environment as realistic. Nonimmersive virtual environments often consist of images and sound presented on a computer without specialized equipment. These less-immersive virtual worlds leave users aware of their real-world surroundings. Alternatively, immersive virtual environments generally incorporate specialized equipment like head-mounted displays (HMDs) or haptic devices to help a user feel as if he or she is physically present in the virtual environment.

Immersion is an important aspect of the *fidelity* of a system. In the context of simulations and virtual environments, the term *fidelity* is used to refer to the degree of similarity between a virtual and a real environment [48]. More immersive environments tend to be higher in fidelity than less immersive environments. However, higher fidelity does not necessarily translate to better learning; rather, fidelity, learning objectives, and level of expertise should be carefully matched. It might seem intuitive that increasing fidelity improves training experiences, but that is not always the case. The ‘Alessi hypothesis’ suggests that there is a certain point for which increasing fidelity no longer improves training at the same rate [49]. Further, lower fidelity might have advantages for novice learners for cases in which higher complexity and more details might compete for the learner’s limited attention.

Wickens and Hollands [50] indicated that three things should be considered when developing a new training system: which device or procedure is cheapest, provides the longest retention, and creates the best learning in the shortest time period? To answer these questions, it is necessary to consider the essential components of the task(s) and the level of fidelity, including immersion, needed to meet training goals. Hays and Singer [6] similarly emphasized that ‘the real issue is to replicate those parts of the task situation which are necessary for learning to perform the task’.

As learners gain expertise and their basic skills approach automaticity, higher-fidelity VR simulation might be employed for more complete training experiences. Higher fidelity likely engenders a higher sense of presence, which can potentially make VR training more effective. Although consensus about a link between presence and learning is lacking, presence in virtual learning environments has been associated with outcomes related to an individual’s ability and motivation to learn [51]. It might be the case that learning is better when VR contains didactic components such as artificial guidance or feedback [52], which lowers cognitive fidelity and decreases presence but increases the utility of the system as a training tool.

Although high fidelity and immersion of virtual environments are not always required for effective training, a high degree of realism is necessary to meet high-level training goals relevant to true surgical expertise. Training theories suggest that a transfer task must share similar structural elements to a training task for training to be most effective. The military refers to this concept as ‘train how you fight’ [42]. That is, a higher level of fidelity of a virtual system is required to address the training of rule- and knowledge-based behavior, whereas skill-based behavior can be addressed with low-fidelity trainers. Surgical trainees have limited opportunities to practice genuine procedures on living patients, but immersive virtual environments can help bridge the gap and create more learning experiences with complex tasks in stressful environments.

### Comparing presence and immersion

The terms *immersion* and *presence* have been used in various ways within various disciplines [53-56]. Slater et al. [57] separated the concepts of immersion and presence by defining immersion as ‘a description of the capabilities of a system,’ whereas presence ‘characterizes the response of participants to the system’. The scientific community, if not the general technology community, has adopted these general definitions when applied to research on virtual environments. The user’s sense of presence is essentially mediated by technology capabilities and design choices of the virtual environment. Therefore, although the two concepts are highly related, aspects of immersion refer to quantifiable features of the technology whereas aspects of presence describe a subjective, qualitative experience of the user. Some factors that govern immersion, and consequently presence, include the following: the level of interactivity a user has with the virtual world, the modes of interaction and control, the field of view of the display, the update rate of the display, and isolation from the real world. The implementation of immersive virtual reality for surgical training that facilitates skill-, rule-, and knowledge-based behavior also follows the principles of improving presence and immersion.

#### Presence

Presence is considered the defining experience of virtual environments [58], meaning an ultimate goal in the design of immersive virtual environments should be to foster a sense of presence in users. Studies tend to show positive relationships between a user’s sense of presence and a user’s experience in a virtual environment. For example, an effect of presence has been found in performance [59,60], emotional reactions [61], and brand recognition and purchasing behavior [62]. Although users are consciously aware that they are not physically located in the virtual space, they might think and behave as if they are.

Recall that Slater et al. [57] defined presence as a user’s response to a system. Presence is highly dependent on a person’s psychological state while interacting with a virtual world. Accordingly, Witmer and Singer [63] referred to presence using psychological terminology, defining presence as a ‘normal awareness phenomenon that requires directed attention’. In a more expansive description, Lee [53] defined presence as ‘a psychological state in which the virtuality of experience is unnoticed’ and divided presence into three domains based on how humans experience the world: physical, social, and self.

Lee’s definition of sense of presence highlights the user’s awareness of separation (or lack of separation) between the physical and virtual world. Factors that influence presence tend to be similar to those that influence immersion, since the two are closely related concepts. Several researchers have attempted to define factors that contribute to a sense of presence [63-65]. Witmer and Singer [63] classified the qualities of virtual environments that influence presence into four types: control factors, sensory factors, distraction factors, and realism factors described in Table 2.

**Table 2:** **Factors that influence presence (Witmer and Singer [** 63 **])**

	**Examples**
Control factors	• Degree of control
	• Immediacy of control
	• Anticipation of events
	• Mode of control
	• Physical environment modifiability
Sensory factors	• Environment richness
	• Multimodal presentation
	• Consistency of multimodal information
	• Degree of movement perception
Distraction factors	• Isolation from the physical world
	• Selective attention
	• Interface awareness
Realism factors	• Scene realism
	• Information consistency with objective world
	• Meaningfulness of experience
	• Separation anxiety/disorientation

One sensory factor in particular, multimodal presentation, is important to consider if high realism is a goal of the virtual environment. Multimodal virtual environments better enable a sense of presence relative to single-sensory technologies, perhaps because information from multiple coordinating senses decreases mental processing time and replicates real-world perception [66,67]. Our daily experiences are often multimodal by nature. Simply reaching out to pick up an object involves input from visual, haptic, and vestibular systems [68]. Communicating with other people is also accomplished through corresponding audio and visual cues: the sound of a person’s voice, the image of lip movements, and the image of gestures. Thus, including multimodal components to a virtual environment, using factors like auditory cues and haptic feedback, can help enhance presence.

A final consideration for strengthening users’ presence is to reduce distractions to the virtual experience, such as by using physical dividers and headphones. A common distraction is simulator sickness, presenting with symptoms similar to other kinds of motion sickness. Symptoms like nausea, oculomotor disturbances (such as eye strain), and disorientation [69] can occur when human sensory systems conflict, as in the case of illusory motion induced by virtual environments [70] or mismatches between stereoscopy and other depth cues [71]. Simulator sickness can be assessed using pre- and post-exposure completion of the Simulator Sickness Questionnaire (SSQ) [69], which measures the 27 symptoms associated with simulator sickness. To avoid extreme simulator sickness, exposure should be limited. Additionally, users’ tendency for motion sickness might be screened and those individuals with high tendency for motion sickness might be discouraged from taking part in virtual reality research.

#### Presence measures

Presence refers to a psychological experience and thus cannot readily be measured directly. However, researchers have used subjective, physiological, and objective measures to assess users’ sense of presence. Because presence measures are indirect, multiple corresponding measures are preferred in presence research.

First, subjective measures based on questionnaires may be used. There are several validated presence questionnaires. Witmer and Singer’s [72] Presence Questionnaire consists of 7-point rating scales with high reliability. Other common subjective presence questionnaires are Schubert et al. [73] Ingroup Presence Questionnaire (IPQ) and Lessiter et al. [74] ITC Sense of Presence Inventory (ITC-SOPI). Although subjective questionnaires are easy to distribute and use, there are a few associated disadvantages. Users might find it difficult to rate their experience because presence is not a readily understood concept by most of the general public [75]. Further, as with all subjective research methods, users might be subject to biases when considering their responses.

Second, physiological measures may be used to infer presence. If a person’s physiological response in a virtual environment is equivalent to real environments, this indicates a high level of presence. An advantage of physiological measures is that they are continuous, enabling an indication of how levels of presence change over time while interacting within a virtual environment [76]. Barfield and Weghorst [77] specifically suggested using measurements of heart rate, pupil dilation, blink responses, and muscle tension. Meehan et al. [78] concluded after using measures of heart rate, skin conductance, and skin temperature as indicators of presence in a virtual environment that heart rate response provided the best assessment. A disadvantage to physiological measures is that they are related to physiological arousal in general and not presence directly. More recently, researchers have used neuroimaging techniques like fMRI [79], EEG [80-82], and TCD [83] to assess brain activity linked to presence in immersive virtual reality. The Emotiv EPOC headset also enables a portable, low-cost method for inferring presence from brain activity [84].

Finally, several creative attempts have been made to use objective measures of presence based on user performance or behavior. For example, Slater and Usoh [65] examined presence by examining participant reactions to simulated objects flying towards their head. Presence is also thought to be a factor of attentional resource allocation [85], meaning increased presence is a result of increased attention. Therefore, performance on a secondary task performed concurrently in the real world might be used to infer an individual’s level of spare attention [77]. However, a drawback to this approach is that a secondary task located outside the virtual world might in itself be distracting and lower presence.

### Physical immersion and devices

When we interact with a virtual world, we often experience a sense of being in that environment despite being located in the physical world. This experience of ‘being there’ tends to be more powerful in immersive environments, such as three-dimensional interactive games, than in less immersive contexts like books or movies [86], thus particularly highlighting the power of the immersive virtual reality environments. The ability to place a surgeon-in-training into a realistic virtual environment, rendering the feeling of ‘being there’ will allow us the ability to custom design training scenarios providing skill-, rule-, and knowledge-based learning.

When selecting technology to create an immersive virtual experience for surgical training, it is important to consider factors that influence the system’s level of immersion and the user’s sense of presence as they relate to the goals of the surgical VR system. How immersive must the equipment be to meet these goals? What sensory systems need to be included in the experience, and to what level of fidelity? To answer these questions, it is important to first understand how hardware and design choices for immersive VR can influence the user’s ability to suspend disbelief and behave as if they are located in the virtual world. Since presence and immersion are so closely related, by using the appropriate technology to enhance immersion, we should be able to enhance the sensation of presence to eventually elevate the level of surgical learning by means of immersive virtual environments.

#### Haptics

Haptic systems or haptics are a part of immersive virtual reality systems that interacts with the user’s sense of touch [87]. Research in haptic feedback has been done since the mid-1900s but has not been able to handle producing believable sensory information at a reasonable cost until recently [88]. Westebring-Van Der Putten et al. highlighted in great detail the importance of haptics in open, minimally invasive robotic, minimally invasive, and VR surgery [89]. In particular, by enhancing physical immersion using haptics, we are creating the sense of presence by means of enhancing the control factors, one of the four factors as detailed by Witmer and Singer [63]. Thus, in order to provide the best available physical immersion, it is critical to thoroughly understand the nature of haptic technology available for surgical simulations in particular.

Haptics is broken down into two main categories, tactile perception and kinesthetic perception [89]. Tactile perception consists of pressure, vibration, and texture. The human body perceives these tactile perceptions through the receptors in our skin. Kinesthetic perception consists of movements and forces. These attributes are perceived through the muscles, tendons, and joints in the body. Regenbrecht et al. [90] stated that presence has three aspects: spatial presence, involvement, and realness of the virtual environment. In order to create the sense of presence, the user must experience all three aspects. Incorporating a haptic system into an immersive virtual reality system will allow the user to experience the realness of the virtual environment. The textures and forces that are present in virtual environment would replicate what the user would feel in the real world, and these forces and textures may be relayed to the user through haptic interfaces. Haptic systems are categorized into four categories: point-based feedback, exoskeletons, wearable systems, and locomotive systems.

##### Point based

Point-based haptic devices are focused on giving the user feedback at one single point. These devices are versatile and can be used as a mouse for the computer or integrated into a virtual reality system. Point-based haptic devices have been successfully implemented into a variety of surgical simulation environments. The most popular point-based product available is the family of haptic devices from Geomagic, in particular the Geomagic Touch (previously known as the Phantom Omni) [91]. It is a serial link mechanism and allows for 6 degrees of freedom (DOF) of motion for the user. The user holds onto a pen-shaped handle, which is used to control the simulation and the location of force feedback to the user. Another example of a point-based haptic device is the Novint Falcon (Novint Technologies, Rockville Centre, NY, USA) which operates using a parallel link mechanism [92].

Despite the availability of many devices that may be suitable for a variety of surgical simulations, there are many limitations these devices have when it specifically relates to surgical simulations. With regard to a given simulation scenario, there are specific needs for degrees of freedom for each hand, force resolution, force bandwidth, minimal device impedance (high transparency), and workspace. These procedure-specific demands placed upon haptic hardware make it near impossible to have a single device that fulfills all criteria. Furthermore, point-based haptic devices provide the user with only force feedback related to the general shape and size of the virtual object, but not the texture and surface details.

The lack of tactile feedback available on kinesthetic force feedback devices has been recognized as shortcoming in the available products on the market. To begin addressing the issue, the Omega.7 from Force Dimensions (Nyon, Switzerland) offers a slight improvement over other point-based kinesthetic-only devices [93]. The Omega.7 uses the same setup as the Falcon with a parallel manipulator that connects to a single point; however, the Omega.7 has a handgrip that offers grasping capabilities. The user can use their index finger to control a gripping device. It provides for 7 DOF with 3 DOF force feedback to the handgrip and 1 degree of freedom force feedback as the gripping interaction [94].

Garcia-Hernandez et al. tested the improvements that tactile feedback would make to the Omega.7 System by adding a tactile display to the Omega.7 grip with a custom-built hand rest. The hand rest allowed the user to place their index finger pad on the tactile display. The tactile display consisted of a 4 × 4 grid of pins, which would protrude when the simulation or a robot detected a displacement. These pins would create a tactile display, which would allow the user to feel the texture and details on the surface. Another product from Force Dimensions is the Sigma.7 [95]. The Sigma.7 was specifically designed for medical and aerospace procedures in tandem with dexterous robots. The Sigma.7 offers 7 DOF and provides 6 DOF of force and torque feedback to the user.

In order to overcome the inherent problem of friction in a variety of haptic devices, magnetic levitation-based systems were developed. Such systems work by providing force feedback to the user holding onto a handle, which levitates within a magnetic field, which is then controlled by controlling/shimming the magnetic field to impact the levitating metallic handle. These devices are favorable because they provide no static friction and no mechanical backlash and have high position accuracy and resolution. The first commercially available magnetic levitation-based haptic device was called the Maglev 200 developed by Butterfly Haptics (Pittsburgh, PA, USA) [96]. The inherent problem with such a device is the high computational cost for determining the magnetic control and instability of the body dynamics while in the magnetic field due to the presence of a human in the system. Last, the device only provided a 14° conical workspace which in regard to surgery is limiting. Similar technologies were developed by Energid Corporation, which built an untethered magnetic haptic feedback system [97]. However, the device was shown to have a 1.5-Hz bandwidth, which was not enough to perform real-time haptic rendering.

The above technologies are mechanical devices, employing actuators (mechanical or magnetic) to drive an object to eventually affect a respective part of the human user to provide force feedback. A new category of nonmechanical noncontact force feedback devices has been developed, employing the principles of ultrasound. Ultrasonic phased arrays are controlled to exert a mechanical wave that travels through the air, displacing the air, creating a pressure difference, which eventually interacts with the user to impart a force. To increase the intensity of the feedback, multiple waves are controlled to arrive at the desired location simultaneously. This technique allows the creation of one or multiple focal points of force feedback. Carter et al. developed UltraHaptics, an ultrasound-based haptic device to render multiple points of discrete force feedback [98]. They were able to statistically show that the smallest detectable separation between two focal points was 2 cm. In terms of haptics, depending on the location of force feedback, the device could be viable or not, for example, the two-point discrimination for the palm is below 3 mm [99].

##### Exoskeleton

An exoskeleton is a device that is worn on the exterior of the user and is attached to the user’s body. Exoskeletons have the benefit of being able to generate much higher ranges of force feedback for multiple joints at the same time, in contrast to point-based devices which act on a single point at a time. Most exoskeletons are stationary and allow for large forces to be generated without a strict size or weight constraint. Those properties of exoskeletons lend themselves very well to surgical procedures requiring large-scale motions of the upper limbs. Procedures such as bi-manual palpation, chest compressions, and intubation all have significantly large ranges of motion, applied forces, and restriction of multiple joints. In order to simulate the above-mentioned procedures, exoskeletons can be used to provide realistic force feedback.

Perry et al. developed the 7 DOF upper-limb exoskeleton designed as an assistive technology for neurorehabilitation [100]. The arm was a cable-actuated device with low inertia, high stiffness in the links, backdrivable along with no backlash.

The CyberForce from Immersion Corporation (San Jose, CA, USA) is a system that incorporates a CyberGlove, an armature, and a hand exoskeleton [101]. The CyberGlove tracks the motion of the wrist, hand, and each finger. The CyberForce provides 6 DOF of motion and provides 3 DOF of force feedback. The exoskeleton on the hand provides force feedback to each finger with the use of cables. Immersion Corporation offers a haptic workstation, which incorporates two CyberForce Systems and a head-mounted display.

The X-Arm 2 [102] and ARMin [103] are both exoskeletons with the focus on providing the user with the most force and torque feedback. The X-Arm 2 provides force feedback to the shoulder, elbow, and wrist. It also provides torque feedback to the forearm and the wrist. The torque feedback may provide force feedback from a simulation of turning a dial on an axle. The arm of the exoskeleton attaches to the chest of the user with a lightweight vest. This device makes physical manipulation of objects in the simulation believable and real with the use of both force and torque feedback. The ARMin also provides force feedback to the shoulder, elbow, and wrist. In addition, it provides torque feedback to the forearm and wrist as well. The ARMin is a stationary device that is attached to the user. The X-ARM 2 and the ARMin allow for full arm movement, force feedback, and torque feedback, which would enhance the sense of presence of the user.

The haptic telexistence exoskeleton created by Sato et al. [104] is a combination of force feedback and tactile feedback to the fingers. The exoskeleton hand is attached to the wrist of the user. There are photoreflectors in the fingertips of the master hand, which detect the position and force of the finger. The force feedback is applied to each of the fingers, and a tactile feedback is applied through the electrotactile display. These electrotactile displays send electric currents to the finger pads of the user to stimulate the vibration and pressure receptors in the skin. The telexistence exoskeleton allows for natural movements of the hands, without having the feeling of wearing anything on the fingers, and provides force and tactile feedback to the user.

A new and emerging device in the haptic field is the Novint Xio (Novint Technologies, Rockville Centre, NY, USA) [105]. This device is currently designed for military simulation games but with application in a wide variety of immersive virtual reality situations. This device consists of an exoskeleton sleeve that goes on the arm, a vest, a backpack, and a head-mounted display. The exoskeleton provides force feedback to the arm and simulates recoil from a military weapon. The vest has vibration generators, which will simulate being hit by a shock wave or an object in the chest. There are also accelerometers in the vest and backpack, which sense if the user is running or walking. The feedback given by the exoskeleton, vest, and feeling of movement from the ground gives the user a truly immersive experience with haptic feedback in different areas of the body.

Despite the wide variety of exoskeletons available on the market including devices developed in research labs, there are significant drawbacks of these systems. The large size of the devices creates significant inertia and inhibits the accurate rendering of tissue impedance to the user wearing the device. Since surgeons performing procedures such as palpation, chest compression, and intubation rely so deeply on the static and dynamic response of their patient’s tissue, inaccurate rendering of those tissue responses through kinesthetic force feedback will detrimentally affect learning. Poor rendering of tissue force feedback disrupts the very first level of learning, the skill-based learning. Thus, future development of exoskeleton devices need to explore materials, actuation technologies, and control algorithms that can sufficiently mitigate the inherent dynamics of such large systems so as to improve the transmission of kinesthetic force feedback to the user.

##### Wearable

Wearable haptic devices are relatively small devices that are worn by the user, typically on the hands as a glove. The benefit of wearable haptic devices is that they can be used with the natural motions of the user without being weighed down by a stationary exoskeleton or bulky device. The use of natural motions would allow for a better immersive experience because it would allow the user to tap into their muscle memory and past experiences [106]. Surgical procedures requiring significant manipulation using primarily the fingers are particularly well suited for the use of wearable haptic devices. However, our fingers possess some of the highest density of mechanoreceptors, making force feedback rendering to the fingers that much more important. Despite wearable haptics being smaller in size, potentially possessing lower inertia, greater transparency, and better suited overall dynamics in comparison to point-based and exoskeleton haptic devices, there are major issues with degree of freedom, primarily in finger-based wearable haptics. Due to mechanical constraints, most devices only provide force feedback in finger extension and flexion, with none in adduction of abduction. Finger manipulation-based tasks in surgery do not solely use one type of motion; mostly, they are a combination of multiple. This inherent complexity in surgery-related finger manipulation makes wearable haptics a challenging category of haptics.

The Master II created at Rutgers is an example of a wearable haptic device [107]. This device has a rubber glove, which the user wears, and pneumatic cylinders, which are connected to the fingertips. This device provides force feedback to each finger. This device is relatively simple and only gives force feedback to the fingers. The force feedback to the hand and arm is lacking.

The CyberGrasp is a more sophisticated glove that is offered by CyberGlove Systems [108]. The CyberGrasp is a portion of what is used in the CyberForce system. The glove tracks motion of each finger and the hand. The exoskeleton has cables that attach to the fingertips of each finger. To produce force feedback in each finger, a motor would apply a force onto the cable.

Magnenat-Thalmann et al. [109] created a system to provide users with the ability to feel texture of fabrics. Their system incorporates a glove, stereoscopic glasses, and a monitor. The glove has a vibration generator in the index finger and thumb. These vibration generators were used to create texture for the fingers. This technology paired up with an exoskeleton arm or force feedback device may provide a more fulfilling immersive experience for the user.

Prattichizzo et al. [110] came up with a device to make haptic systems more wearable. The device that was designed was a fingertip haptic device. It consists of three motors, three wires, and a force feedback plate. The plate is attached to the motors through the three wires at three corners. The device provides force feedback by the motors pulling the wires and providing a force in the force plate. The force plate applies the force on the fingerpad of the user and creates a feeling of pressure and force. This is one of the smallest haptic devices and allows for unrestricted and free movement of the hands and fingers.

##### Locomotive

A locomotive haptic system is a full-body experience where the device simulates real walking as if the user is navigating through the simulation. The experience allows the user to interaction and resistance forces from walking as if they were in the virtual environment. These devices have traditionally been designed and developed for military and gaming experiences requiring the need to travel through the virtual environment. They can be particularly useful in medical crisis simulation environments where team interactions are critical, such as in the emergency room. Such devices are meant to enhance physical immersion in environments where the goal is to learn a knowledge-based behavior, which as mentioned before can be ER-like environments.

The Tradport is an example of a locomotive haptic system. The Tradport consists of a CAVE visual display, a treadmill, and body harness. This system allows the user to walk around the simulation on the treadmill. This immersive experience gives the user force feedback at his feet, and it simulates real walking through a simulation.

The Virtuix Omni [111] is an omnidirectional treadmill where the user can control their avatar in the simulation just by running and turning in the device. The user wears a low friction shoe which allows the user to run in the concave base. The Virtuix Omni paired with a head-mounted display would allow the user to navigate and be immersed in a virtual environment. Virtualizer VR from Cyberith (Herzogenburg, Austria) offers full-body motion control [112]. The rig has an omnidirectional treadmill which allows the user to run and walk through a virtual simulation. The rig also allows the user to be able to sit down if the avatar in the simulation is sitting. The pillars on the rig track the vertical motions of the user. This could track jumping, couching, and sitting movements of the user. The rig is compatible with head-mounted displays to be immersed in the environment. These locomotive haptic devices allow for the full-body experience of navigating in the virtual environment such as an ER.

#### Motion/control

To create a sense of presence, the user must feel that they are in the virtual environment. The three parts of creating a sense of presence are spatial presence, involvement, and realness/naturalness of the virtual environment [90]. The user’s inputs play a large part in each portion of presence. As per Witmer and Singer’s [63] model of presence, the use of motion and control technology can enhance the quality of the virtual environment by controlling sensory factors. Since the user’s inputs are ways that the user can interact with virtual reality software to control something in the virtual environment, it seeks to improve presence. It is very critical to accurately capture the motions of the user in a surgical simulation environment. Since a component of surgical learning is motor learning, any inaccurate or sub-par depiction of the user’s motion in the simulation can disrupt such motor learning.

An ordinary example of a user input would be a mouse and keyboard being used to type or navigate through the operating system. The same tools as mouse and keyboard can be used in an immersive virtual reality simulation but would not yield the same level of presence as more natural interactions. A sense of presence is increased when the user begins to see that their own movements are emulated in the simulation. Therefore, the naturalness of the input for the user has a direct correlation to the sense of presence. The technology that is being used for inputs in immersive virtual reality can be categorized under these categories: optical trackers, acoustic trackers, mechanical trackers, magnetic trackers, inertial trackers, data gloves, and eye trackers [113].

##### Optical trackers

Optical trackers are more widely used in high-end motion picture production, but this technique can be used for immersive virtual reality. These devices incorporate a camera system to track the motion. Optical trackers fall under three categories: beacon trackers, pattern recognition, and laser ranging [113]. Beacon trackers use markers placed on the user to track the motions of the user. These markers are usually LEDs or reflective materials. The camera tracks the position of the markers. With an array of markers, the orientation of the body parts may also be determined. The SELSPOT is an example of a beacon tracker [114]. It has 30 infrared LEDs that are tracked by a camera. The OPTOTRAK 3020 (Northern Digital Inc., Waterloo, Ontario, Canada) is another device that uses the same principle [115]. The system can track up to 256 infrared light pulses from a LED. The FlashPoint Model 3000 (Image Guided Surgery Technology Inc., Boulder, CO, USA) is a system that uses infrared LEDs and can track up to 360 markers per second. These two systems have been tested for computer-aided surgery capabilities. The DynaSight Sensor from Origin Instruments (Grand Prairie, TX, USA) uses an infrared camera to track markers that are placed on the user [116].

The Microsoft Kinect is an optical tracker which has been introduced more recently to the market [117]. The Kinect has a depth sensor, color camera, and a four microphone array that is used for motion tracking. The depth sensor consists of an infrared projector and an infrared camera. The projector projects a pattern which is recorded with the camera. The difference in the pattern from the original is used to determine the depth of the user. This depth image is then used to interpret the body parts and the joints. This interpreted information is used to construct a skeleton of the user. The Microsoft Kinect due to its small form factor, accuracy, high frame rate, and low cost has now become a product of choice for surgical simulation environment developers.

The Leap Motion tracking device is a recent product development for optical hand tracking [118]. It is a small compact motion controller that senses natural motions of the hand, allowing motions such as pointing, waving, reaching, and grasping.

##### Acoustic trackers

There are two types of acoustic trackers: time of flight and phase coherent [113]. The time of flight acoustic trackers has an emitter and at least three receiver pairs. The emitter produces a sound wave which is then picked up by the receivers. The time of flight determines how far each receiver is from the emitter. Then, with triangulation, you can determine the position of the object being tracked. The phase-coherent acoustic tracker uses the same setup as the time of flight tracker. However, instead of using time of flight, the difference in phase is used to determine the distance from the emitter. Acoustic trackers allow for a relatively inexpensive way to track objects. However, the acoustic tracking has issues with line of sight and is easily distorted with acoustic interference from echoes or other noise.

##### Mechanical trackers

Mechanical trackers use physical linkage and joints to track the position and orientation of an object. In point-based mechanical trackers, the angles and lengths of the links are measured to determine the position of the object being tracked. Mechanical tracking can also be used in exoskeletons. Exoskeletons have links that are proportional to the body part that it is being worn. The exoskeleton can track the angles of each joint to be used to track the position and orientation of the entire body part and all its joints. The use of physical connections to measure positioning and orientation makes mechanical trackers very accurate and have the ability to provide force feedback if actuated. However, the system may be cumbersome and have limited reach, which makes the working area small and may restrict some of the movements of the user. Such systems are only feasible to be used when used in conjunction with haptics when a specific motion of the user is to be captured.

##### Magnetic tracker

Magnetic trackers are devices that use magnetic fields to find the position and orientation of an object. The system consists of one emitter which generates the magnetic field and receivers that collect measurements on about the magnetic field. The data collected by the receiver is then interpreted to determine the position and orientation of the receiver. The receiver can be placed on the parts of the body that are to be tracked for motion and orientation. Polhemus is one of the leading motion tracking companies. They produce multiple magnetic trackers including Fastrak, IsoTrak II, Inside Trak, Ultratrak, Patriot, Liberty Latus, and G4 [119]. These devices have markers that would be strapped on to the user through Velcro or attached to other positioning devices. Polhemus devices use AC electromagnetic pulses for the emitter. The Patriot, Liberty Latus, and the G4 send the magnetic information wirelessly to the emitter to be interpreted. Flock of Birds by Ascension (Shelburne, VT, USA) is another magnetic tracking system that allows for 6 DOF of motion tracking [120]. Flock of Birds consists of an emitter which uses DC pulses to create a magnetic field. The PC/BIRD and SpacePad both follow the same setup as the Polhemus and Flock of Birds devices. Sixense offers two magnetic trackers for immersive virtual reality [121]. The Sixense Razer Hydra (Sixense, San Francisco, CA, USA) is a ‘nunchuck’ controller that is tracked by a base that emits magnetic waves. The hydra tracks the orientation and position of the hands. The Sixense STEM System (Sixense, San Francisco, CA, USA) is a more versatile magnetic tracker. The STEM System includes five tracking points that can be attached to different parts of the body or used in the hand tracking devices. The base of the STEM System emits electromagnetic fields to track the tracking points. The orientation and position of the points will be tracked.

Magnetic trackers are small and light and can be attached to different parts of the body. In addition, they do not have a line of sight issue and can be obstructed from the emitter. However, the further you are from the emitter the less accurate the receiver is. External magnetic fields and ferromagnetic objects will also distort the receiver’s accuracy. Magnetic trackers are versatile and offer tracking of the user’s natural movements.

##### Inertial trackers

Inertial trackers are devices that consist of gyroscopes or accelerometers that track the motion of an object [122]. They are commercially readily available and inexpensive to manufacture and have thus made their way into a variety of products requiring orientation tracking. The MotionPak is an inertial tracker by Systron Donner Inertial (Concord, CA, USA) and is an inertial sensor that can be strapped onto users to track 6 DOF [123]. The MotionPak utilizes a vibrating quartz tuning fork inside the device to sense angular velocity. Control VR is a whole-body tracker that utilizes inertial trackers that incorporate a gyroscope, accelerometer, and magnetometers. The user aligns their body to a virtual skeleton facing magnetic north. The device tracks the person’s chest, waist, upper arm, forearm, and hands. The hands are tracked through gloves that incorporate inertial sensors. The gloves are able to track the flexion of each individual finger. The Xsens MVN (Xsens, Culver City, CA, USA) is a full-body tracking suit that utilizes 17 inertial trackers in addition to head and hand tracking [124]. These trackers are accurate and provide high update rates; however, systems requiring the user to wear body suits can be cumbersome and undesirably interfere with the users’ interactions while in the surgical virtual environment. Minimizing distraction factors as per Witmer and Singer’s model of presence will positively impact the quality of the immersive environment.

##### Data glove

Data gloves are devices that can detect the joint angles in each finger. They allow the user to use the grip motion or any hand gesture in the simulation. This increases the user’s sense of presence since all degrees of freedom of the user’s hand are being captured. The first commercial data glove was developed by VPL who launched the DataGlove in 1989, a fiber-optic data glove which sensed the flexing of the finger [125]. The CyberGlove III from CyberGlove Systems (San Jose, CA, USA) is a data glove that houses up to 22 sensors in the glove [126]. The sensors sense flexion and abduction of each finger and the palm arch. The CyberGlove paired with another type of tracker can give the hand orientation relative to the rest of the body.

##### Eye trackers

There are four types of eye tracking technologies: limbus tracking, image tracking, electro-oculography, and corneal reflection. Limbus tracking devices use infrared LEDs and photo-transistors to track the gaze through the reflection off of the iris and sclera of the infrared light off the eye. The image tracking devices use a camera to detect the gaze direction. Electro-oculography devices use electrodes placed near the eye to detect electrical potential between the cornea and retina to determine eye movement and gaze. Corneal reflection devices utilize the reflection of light off the convex cornea surface. The BioMuse (BioControl Systems, Bellevue, WA, USA) is an electro-oculography device that is available for eye tracking. The BioMuse allows for eye tracking; however, the vertical movements become unreliable due to the reflex motion of blinking [127]. The Headhunter Head and Eye Tracking System (ISCAN, Inc., Woburn, MA, USA) is a limbus tracking device [128]. It tracks the reflection of infrared light off the cornea or pupil. The EyeGaze System (LC Technologies, Inc., Fairfax, VA, USA) is also an image tracking [129]. The center of the lens of the eye is illuminated with IR light. A camera then processes the reflection off of the eye and determines the gaze direction. The Dual Purkinje Image Eye Tracker (Fourward Optical Technologies, Inc., Buena Vista, VA, USA) utilizes the Purkinje image to determine eye gaze. The Purkinje 1 reflection is compared to the Purkinje 4 reflection to determine the gaze direction.

Eye tracking systems possess the ability to provide two important facets of information regarding gaze. First, they serve as input devices for the user’s direction of sight and allow the scenes to be rendered accordingly. Secondly, with regard to surgical simulations, they provide another set of performance information when in a scenario meant to impact knowledge-based behavior. In a complex scenario with multiple events occurring simultaneously, by tracking the gaze of the user, it can be determined where the user was looking during the course of the complex scenario. Questions such as, was the user looking at the correct area during the course of the simulation? Did the user give critical areas enough attention? Such questions regarding attention dedication in an environment laden with multiple simultaneous events allow us to identify how the user performed.

#### Visual

In an immersive virtual reality system, the visual system is one of the most important systems. Bjork and Holopainsen stated that when creating immersion, there is a category called spatial immersion where the user feels like they are inside the simulation and everything appears real [130]. Visual systems are essential in virtual reality systems to put the user in the simulation and give them the sense of spatial immersion. For virtual reality systems, there are mainly two systems that have been widely used.

##### CAVE

The CAVE system is a visual system that has been used widely by immersive virtual reality systems. The CAVE was first developed in 1991 and was showcased in 1992 at SIGGRAPH [131]. The CAVE system is a display that surrounds the user and immerses the user in the environment. The CAVE uses projectors that project the images on the walls and floor of a small room. In this specific CAVE system, the user’s position was tracked to change the images that were projected to fit the perspective of the user. Since then, the CAVE display systems have become more advanced. Corporations that have developed a commercial CAVE system include Barco [132], Mechdyne [133], and Igloovision [134]. The Barco Cave Display offers four, five, or six wall displays including the floor (Barco). This system is capable of stereoscopic projection. Mechdyne produces two versions of the CAVE. The Mechdyne CAVE (Mechdyne Marshalltown, IA, USA) also offers four, five, or six wall displays of a room. The Mechdyne CAVE is also capable of stereoscopic projection. The Mechdyne CAVE 2 is a 360° panoramic screen that consists of 72 three-dimensional LCD panels. The user is surrounded by the display but not below. The Igloo is a dome-shaped display system where the user steps inside the dome which offers 360° of vision. This is achieved with the use of a projector array which is centered at the top of the dome. The IllumiRoom is a project by Microsoft which augments the space around you [135]. This system has the same principle as the CAVE system, but the IllumiRoom uses one wall. CAVE Systems offer a very immersive experience for the user. The downside to a CAVE system is that it is not very versatile and needs to be set up. The biggest limitation for a CAVE system is the amount of space needed for the system to be set up.

##### Head-mounted display

The head-mounted display gives the user an immersive experience with a relatively small amount of space. A head-mounted display is a small display that places the screen directly in front of the eyes. The head-mounted display creates an environment where the surroundings are blocked out and the display covers the user’s field of view.

Sensics offers a wide array of head-mounted displays. Sensics (Columbia, MD, USA) offers zSight 810, dSight, rSight, zSight, zSight - 1920, xSight, and piSight [136]. The Sensics HMDs have a panoramic screen which gives the user ability to also use their peripherals. These HMDs also have the ability to track the users pitch, yaw, and roll. In addition, they can track the acceleration in 3 DOF. The Vuzix VR920 HMD (Vuzix, Rochester, NY, USA) and the Vuzix VR 1200 HMD (Vuzix, Rochester, NY, USA) use two LCD displays [137]. They also have 3 DOF of orientation tracking. Sony also has a line of HMDs, named HMZ, that gives the user an immersive experience. The Sony HMZ-T1 (Sony Corporation, Minato, Tokyo, Japan) gives the user 45° of field of view horizontally and a 720p OLED screen [138]. However, the T1 does not offer head tracking. The newly revealed T3Q device was developed to compete with other head-mounted displays. The T3W is predicted to offer 720p to 1080p to the user. The design of this HMD simulates a 750-in. screen at 62.5 ft away from the user [139]. The T3W has head tracking in order to compete with other head-mounted displays.

The main competitor in the head-mounted display field is the Oculus Rift (Oculus VR, Menlo Park, CA, USA) [140]. The Oculus Rift is an affordable head-mounted display that is geared towards gaming but has immense potential to be applied to surgical simulation environments. The Development Kit 1 (DK1) version of the Rift provides a LED screen which covers 90° field of view horizontally to the user and is capable of stereoscopic video. The Rift also tracks yaw, pitch, and roll. However, it does not track the position of the user’s head. The updated Development Kit 2 (DK2) offers more features. The DK2 model has an OLED screen which covers 90 DOF of the user. The DK2 model has tracking sensors that track the head orientation, position tracking with an infrared camera, gyroscope, accelerometer, and a magnetometer. The magnetometer is used to align the device with a compass in order to reduce drift over time. These sensors help the track head motions that allow the software to correctly simulate the motions to match up with the real world. The position tracking of the DK2 also allows the user to lean in all directions in the simulation. This helps simulate natural motions the person would make.

#### Auditory

The human brain and ears can uniquely locate sounds in a three-dimensional environment [141]. The use of audio in simulation environments serves to further immerse us in the simulation environment. Replicating realistic audio arising from seemingly realistic locations within the environment enhances the spatial presence of the user. This accuracy of audio and simulated region of origin for the audio must be in sync; any discord will contribute to a breaking of the perception of presence. Another powerful tool of auditory feedback is the sensation of vection. Vection is the sensation that one is moving while physically not in motion as a result of the auditory stimulus. Larsson et al. found that with the use of headphones and the shifting of the placement of the sound, vection could be created [142]. This shows that audio could give a person a sense of spatial presence. Another contributor to enhancing presence is isolation. Isolation allows for the user to block off all outside distractions and concentrate on the simulation. This provides the user the ability to be immersed in the environment and gain the sense of presence.

The evolution of audio has gone from one channel mono speakers to eight channel surround sound speakers. The addition of channels allows the speakers to surround the user and offers sound from all directions. The first two channel stereo systems were used in 1934. From there, the evolution of sound continued in 1950 when the first multichannel surround sound was used. Audio has been used for a long time for media and the arts. However, sound has not been specifically altered or enhanced for the use of immersive virtual reality until recently. There are two projects which have used sound in tandem with motion tracking in order to provide an immersive experience. These are the LISTEN Project and the 3-D Audio Project by Microsoft.

The LISTEN Project was used in an art museum. The users in the gallery wore wireless motion-tracked headphones that immersed them in the auditory scene [143]. The position tracking of the headphones allowed each piece to have audio come from them. As you got closer to an object, the audio became louder. This technology could also be used in immersive virtual reality, to provide the sensation of an object/person’s distance from another object/person. Such technologies in collaboration with HMD’s or CAVE systems can produce powerfully immersive systems dedicated to surgical simulation environments.

Microsoft’s project of 3-D Audio also uses motion tracking to give the user a more immersive experience. The user wears a pair of headphones which track the user’s head orientation and motion. The software pinpoints the user’s location and simulates the sound coming from the object at that specific location. The current issue with making sounds localized to a specific point is the variation of the head anatomy and ear shape from person to person. The sound will travel to the ear differently from person to person. This is called the head-related transfer function [144]. The head-related transfer function (HRTF) is a model of how sounds travel through a person’s head and ears. This model allows for the computer software to place the origin of the sound at the most accurate position. The developers have created a way to approximate the user’s HRTF with the use of a Microsoft Kinect. The Kinect creates a model of the user’s shoulders and head shape. Using this information, the system approximates the user’s HRTF by comparing the shoulder and head shape to a database of 250 shapes with HRTF values. This allows for accurate positioning of sounds and creates a more immersive experience. With such low cost and ease of implementation, user-specific auditor feedback with HMD’s is a very real possibility to further enhance presence.

#### Avatars and presence

When users interact with a virtual environment via an avatar, how is the user’s sense of presence impacted? Researchers have reported users feeling a psychological connection with avatars they create [145,56,146]. A realistic representation of the user in a virtual environment, such as an avatar similar in appearance to the user, will likely increase the user’s connection with the character and their sense of presence [147]. Slater and Usoh [65] found positive effects of an avatar on participants’ reports of presence, such as maintaining presence when display problems were encountered.

Immersive virtual worlds also provide a meaningful framework for users’ interaction with others [146]. Aspects of other characters in virtual environments, whether embodied agents representing computers or avatars representing other humans, may also influence a user’s sense of presence in that environment. Copresence is a concept describing how users psychologically perceive themselves as sharing a virtual space with virtual characters [148,149]. For example, Ahn et al. [150] found that presenting virtual humans as life size using a stereoscopic display mode increased aspects of presence compared to presenting the characters in nonstereoscopic conditions or on computer monitors. Such studies suggest that avatar-based team interactions can in fact improve the presence of a user, which in particular are very powerful for creating simulation environments meant to impart knowledge-based learning. Surgeries in operating rooms are not performed in isolation by the surgeon; they are performed with multiple staff and a variety of ambient distractors. Bringing the social interaction aspect of an operating theater into the simulation environment will in effect improve enhance presence and eventually learning, as suggested by Lee’s [53] model of presence.

#### Computational hardware

Virtual reality-based simulation environments are human-in-the-loop systems by nature. All the entities of the virtual environment such as visual rendering effects and tactile feedback should therefore adhere to the constraints of real-time update violation of which could lead to compromised VR immersion. The real-time requirement for the visual feedback is a minimum of 30 Hz while that of the tactile feedback is around 700 Hz for soft contacts and close to 1,000 Hz for stiff contacts. One of the main challenges of building a real-time VR environment is to be able to furnish the required frame rate by performing rapid computations. While faster algorithms could fulfill this requirement to a certain degree, efficient hardware could boost the performance by a considerable extent.

Most of the real-time VR applications relied traditionally on the commodity hardware such as desktops and workstations. The computing power of the central processing units (CPUs) have grown rapidly at the average rate of 52% a year starting in the mid-1980s until around 2002 [151]. Since then, the rate has decreased to around 20% owing to power limitations, memory latency, and limited availability of instruction-level parallelism. Researchers over the years have taken advantage of the parallel capability hardware through parallelizing the algorithms using shared memory parallelism [151,152].

The advent of graphical processing units (GPUs) as a general purpose computing hardware architecture has boosted the performance of the applications enormously. GPUs are inherently parallel with thousands of cores enabling highly parallel processing of instructions. The present-day GPUs can reach a peak performance of up to 1,600 Gflops [153,154] surpassing the parallel CPU architectures. Software architectures such as CUDA and OpenCL have further popularized the GPUs as a general purpose computing hardware platform. Many applications developed for GPUs have achieved orders of magnitude speedups [155-159].

GPU programming at the moment is more involved and is required to be tuned heavily to be able to achieve peak parallel performance. Intel’s Intel® Phi™ architecture addresses. Intel® Phi™ can provide 1.2 teraflops of performance in the best case. Parallelization on Intel® Phi™ requires minimal restructuring of the code and can be optimized using existing profiling tools like Intel® Parallel studio. This platform could benefit the real-time applications since the existing codes can be easily parallelized using well-known parallel software APIs such as p-threads, OpenMP, and MPI.

## Conclusions

While there has been much progress in the development of virtual reality-based technology for learning basic surgical skills, its full potential is yet to be realized in gaining true surgical expertise. Based on Rasmussen’s skill-, rule-, and knowledge-based theory of human behavior, we conclude that immersive virtual reality (iVR) is essential to gain mastery. The path towards iVR is, however, challenging as substantial developments are necessary in the fields of immersive display systems and computational hardware.

Significant advancements, summarized in this paper, have generated enthusiasm that the goal of iVR may not be out of reach. It is already possible to generate interactive simulations with deformable organ models involving force feedback for tens to hundreds of thousands of degrees of freedom. Exponential advances in computing will make real-time computations affordable for environments with increasing complexity involving tens of millions of degrees of freedom in the next 10 years. Quantum computing offers another interesting development that could potentially be a game changer if it can be developed in an efficient manner.

High-fidelity interface devices will, however, remain the major obstacle in the foreseeable future. Effectively recreating sensory input for seamless interactions will require new thoughts and ideas. Direct displays onto the retina and artificial stimulation of the mechanoreceptors and auditory nerves may bring us closer to the goal of iVR.
